# Boolean analysis identifies CD38 as a biomarker of aggressive localized prostate cancer

**DOI:** 10.18632/oncotarget.23973

**Published:** 2018-01-05

**Authors:** Debashis Sahoo, Wei Wei, Heidi Auman, Antonio Hurtado-Coll, Peter R. Carroll, Ladan Fazli, Martin E. Gleave, Daniel W. Lin, Peter S. Nelson, Jeff Simko, Ian M. Thompson, Robin J. Leach, Dean A. Troyer, Lawrence D. True, Jesse K. McKenney, Ziding Feng, James D. Brooks

**Affiliations:** ^1^ Department of Pediatrics and Department of Computer Science and Engineering, University of California San Diego, San Diego, CA, USA; ^2^ The Department of Biostatistics, The University of Texas MD Anderson Cancer Center, Houston, TX, USA; ^3^ Canary Foundation, Canary Center at Stanford, Palo Alto, CA, USA; ^4^ The Prostate Center at Vancouver General Hospital, University of British Columbia, Vancouver, British Columbia, Canada; ^5^ Department of Urology, University of California San Francisco, San Francisco, CA, USA; ^6^ Department of Urology, University of Washington Medical Center, Seattle, WA, USA; ^7^ Division of Human Biology, Fred Hutchinson Cancer Research Center, Seattle, WA, USA; ^8^ Department of Pathology, University of California San Francisco, San Francisco, CA, USA; ^9^ CHRISTUS Medical Center Hospital, San Antonio, Texas, USA; ^10^ Department of Urology, University of Texas Health at San Antonio, San Antonio, TX, USA; ^11^ Eastern Virginia Medical School, Pathology, Microbiology and Molecular Biology, Norfolk, VA, USA; ^12^ Department of Pathology, University of Washington Medical Center, Seattle, WA, USA; ^13^ Department of Pathology, Cleveland Clinic, Cleveland, OH, USA; ^14^ Department of Urology, Stanford University, Stanford, CA, USA

**Keywords:** Prostate cancer, CD38, ARG2, Prognosis, biochemical recurrence

## Abstract

The introduction of serum Prostate Specific Antigen (PSA) testing nearly 30 years ago has been associated with a significant shift towards localized disease and decreased deaths due to prostate cancer. Recognition that PSA testing has caused over diagnosis and over treatment of prostate cancer has generated considerable controversy over its value, and has spurred efforts to identify prognostic biomarkers to distinguish patients who need treatment from those that can be observed. Recent studies show that cancer is heterogeneous and forms a hierarchy of tumor cell populations. We developed a method of identifying prostate cancer differentiation states related to androgen signaling using Boolean logic. Using gene expression data, we identified two markers, CD38 and ARG2, that group prostate cancer into three differentiation states. Cancers with CD38-, ARG2- expression patterns, corresponding to an undifferentiated state, had significantly lower 10-year recurrence-free survival compared to the most differentiated group (CD38+ARG2+). We carried out immunohistochemical (IHC) staining for these two markers in a single institution (Stanford; *n =* 234) and multi-institution (Canary; *n =* 1326) cohorts. IHC staining for CD38 and ARG2 in the Stanford cohort demonstrated that combined expression of CD38 and ARG2 was prognostic. In the Canary cohort, low CD38 protein expression by IHC was significantly associated with recurrence-free survival (RFS), seminal vesicle invasion (SVI), extra-capsular extension (ECE) in univariable analysis. In multivariable analysis, ARG2 and CD38 IHC staining results were not independently associated with RFS, overall survival, or disease-specific survival after adjusting for other factors including SVI, ECE, Gleason score, pre-operative PSA, and surgical margins.

## INTRODUCTION

Although screening and early detection of prostate cancer (PC) has been associated with a drop in prostate cancer specific mortality in the US population and in the European Randomized Study of Prostate Cancer (ERSPC), PSA testing leads to significant over-detection and over-treatment of localized disease [1, 2]. Because of morbidities arising from local therapies such as surgery or radiation therapy and lack of benefit in large randomized trials of PSA screening and surgery in the U.S., the U.S. Preventative Services Task Force had, for 5 years, recommended against PSA testing [3–5]. Recently this recommendation was revised in favor of discussing the risks and benefits of PSA testing after several criticisms of the US trials and a dramatic shift in management of low risk patients to Active Surveillance (AS) [6]. Since 30–50% of men on AS will reclassify to higher volume or higher Gleason score after 5 years of surveillance [7], considerable effort has been devoted to identifying early those men at high risk for reclassification by developing prognostic biomarkers. We have focused on identifying and validating tissue-based immunohistochemical biomarkers of aggressiveness since they can be widely deployed in pathology suites and are relatively inexpensive [8].

Many prognostic biomarkers have been identified by association of their expression with adverse pathological and clinical features including failure after primary therapy. One of the cardinal features of cancer is dedifferentiation, often due to activation of programs active during tissue development [9]. Previously, we have used Boolean logic to discover differentiation programs modulated in bladder and colon cancer [10–12]. Although less is known about the developmental programs in the normal prostate, we applied Boolean logic to identify transcripts co-regulated with AR and its downstream target PSA that are differentially expressed in the luminal and basal compartments of the normal prostate acinus. We chose the AR signaling pathway since it is central to prostate development and function [13, 14].

Normal prostate tissue is comprised of exocrine glands embedded in the prostate stroma containing fibroblasts, variable numbers of inflammatory cells, and smooth muscle cells. Current evidence demonstrates that a subset of the basal cells proliferate and give rise to terminally differentiated luminal secretory cells, suggesting that there are stem-like cells in the basal layer [15–18]. The luminal cells express prostate specific antigen (PSA or KLK3), prostatic acid phosphatase (PAP), androgen receptor (AR), and keratins (K) 8 and 18, while the basal cells express K5 and K14 [19]. Although prostate tumors display a strong luminal phenotype, it is still unclear whether basal cells [20] or luminal cells [21] are the cell of origin for prostate cancer. Hierarchies within the luminal cell types are poorly understood. It has been proposed that graded level of PSA or AR may define different stages of differentiation within luminal compartment [22].

Based on observations in bladder, colon and myeloid cells, we hypothesized that prostate cancer cells may have a path of differentiation in which several genes are turned on at different stages of differentiation [10–12]. We therefore used Boolean logic to identify transcripts modulated between basal and epithelial cells. We identified two genes, CD38 and ARG2 that appear to be associated with prostate cancer differentiation. We tested whether expression level of these transcripts was prognostic using prostate cancer gene expression datasets with associated long-term follow-up. In addition, we tested whether protein expression, measured by IHC, could be used as a clinical biomarker of prognosis.

## RESULTS

### Identification of markers of differentiation in prostate cancer

**Figure 1:**
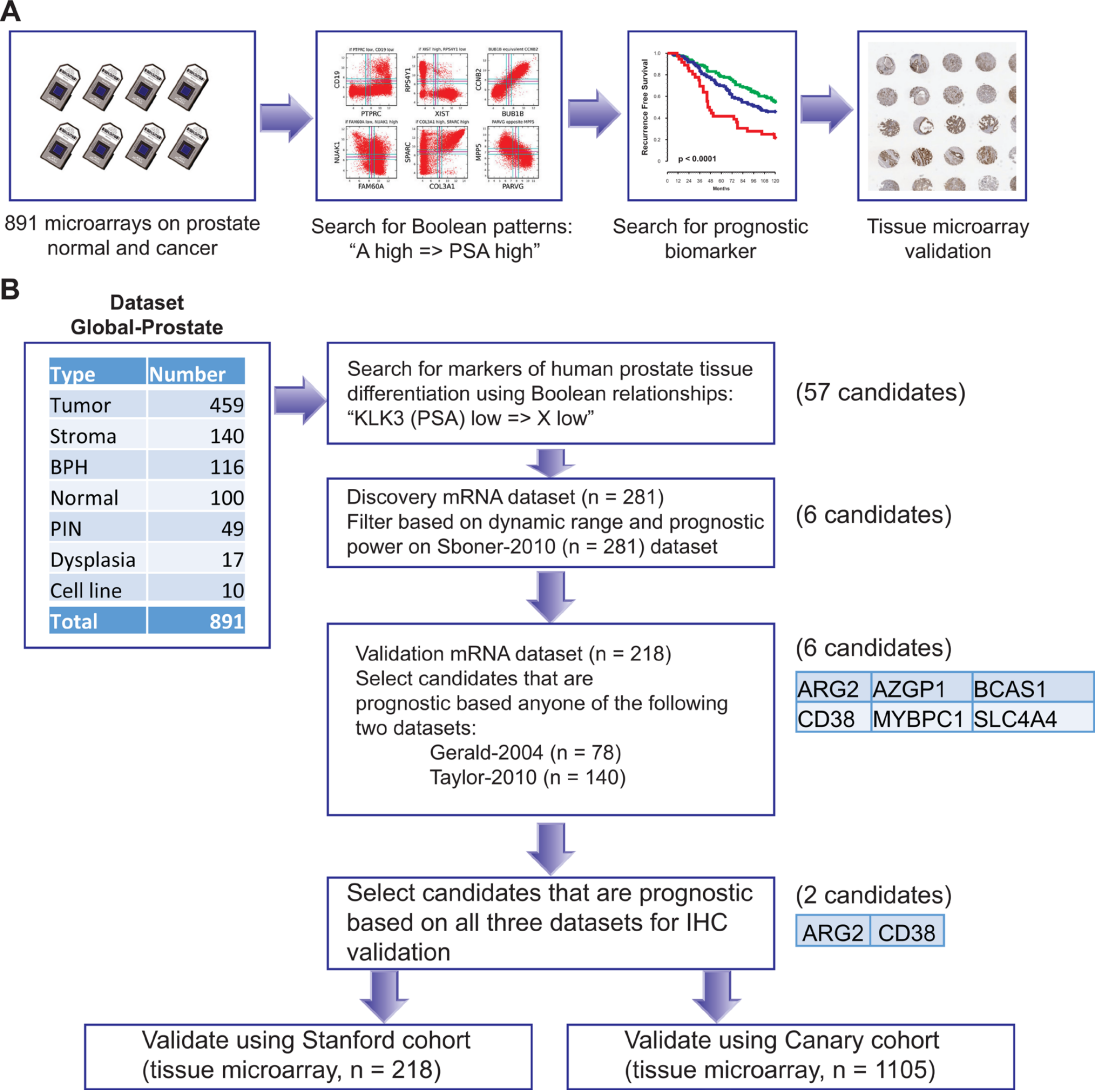
Schematic of experimental design A database of 891 samples (Global-Prostate) related to prostate cancer (*n =* 459), stroma (*n =* 140), benign prostatic hyperplasia (BPH, *n =* 116), normal prostate (*n =* 100), prostatic intraepithelial neoplasia (PIN, *n =* 49), dysplasia (*n =* 17), and cell line (*n =* 10) was created for gene expression analysis. Boolean analysis was performed to identify simple logical patterns between high and low expression values of two genes. A list of 57 candidate genes satisfied “KLK3 (PSA) low => X low” Boolean patterns. These candidates were filtered using high dynamic range and association with outcome using the Sboner-2010 (*n =* 281) as the discovery dataset resulting six candidates. Candidates were evaluated for their association with disease-free survival using two independent validation datasets (Gerald-2004 *n =* 78, Taylor-2010 *n =* 140, Total *n =* 218). All six candidates were significantly (*p <* 0.05) associated with disease-free survival in at least one dataset (Gerald and Taylor). Two of them (CD38 and ARG2) were chosen for immunohistochemical staining-based validation because of availability of good antibodies and their significant (*p <* 0.05) association with disease-free survival in all three datasets.

We assembled a large prostate cancer mRNA dataset (Global-Prostate) for Boolean analysis as shown in Figure 1. The dataset included 459 prostate cancer samples, 140 stroma samples from prostate tissue, 116 benign prostatic hyperplasia, 100 normal prostate tissues, 49 prostatic intraepithelial neoplasia, 17 dysplasia, and 10 cell lines (Total 891 samples). To identify differentiation specific markers, we searched for Boolean expression patterns between PSA (KLK3) and other genes: “KLK3 low => X low” and its counterpart “X high => KLK3 high” (Figure 1A, 1B Supplementary Figure 1). The resulting gene list included 57 transcripts (using s > 10, *p <* 0.01 threshold for the BooleanNet analysis). To filter this list, we assembled three independent publicly available prostate cancer datasets that were annotated for recurrence-free survival and tested whether the transcripts were correlated with recurrence after surgery [23–25]. We used Sboner-2010 as the discovery dataset that revealed 6 transcripts are associated with outcome. One transcript, AZGP1, is a known androgen regulated gene that we have shown previously is an independent predictor of outcome after surgery in the Canary TMA cohort [26]. These candidates were validated on Tayler-2010 and Gerald-2004 dataset where all 6 candidates were associated with outcome in at-least one of the dataset. We chose CD38 and ARG2 because they were associated with outcome in all three datasets and IHC reagents were available that performed well on fixed and paraffin embedded tissues.

**Figure 2:**
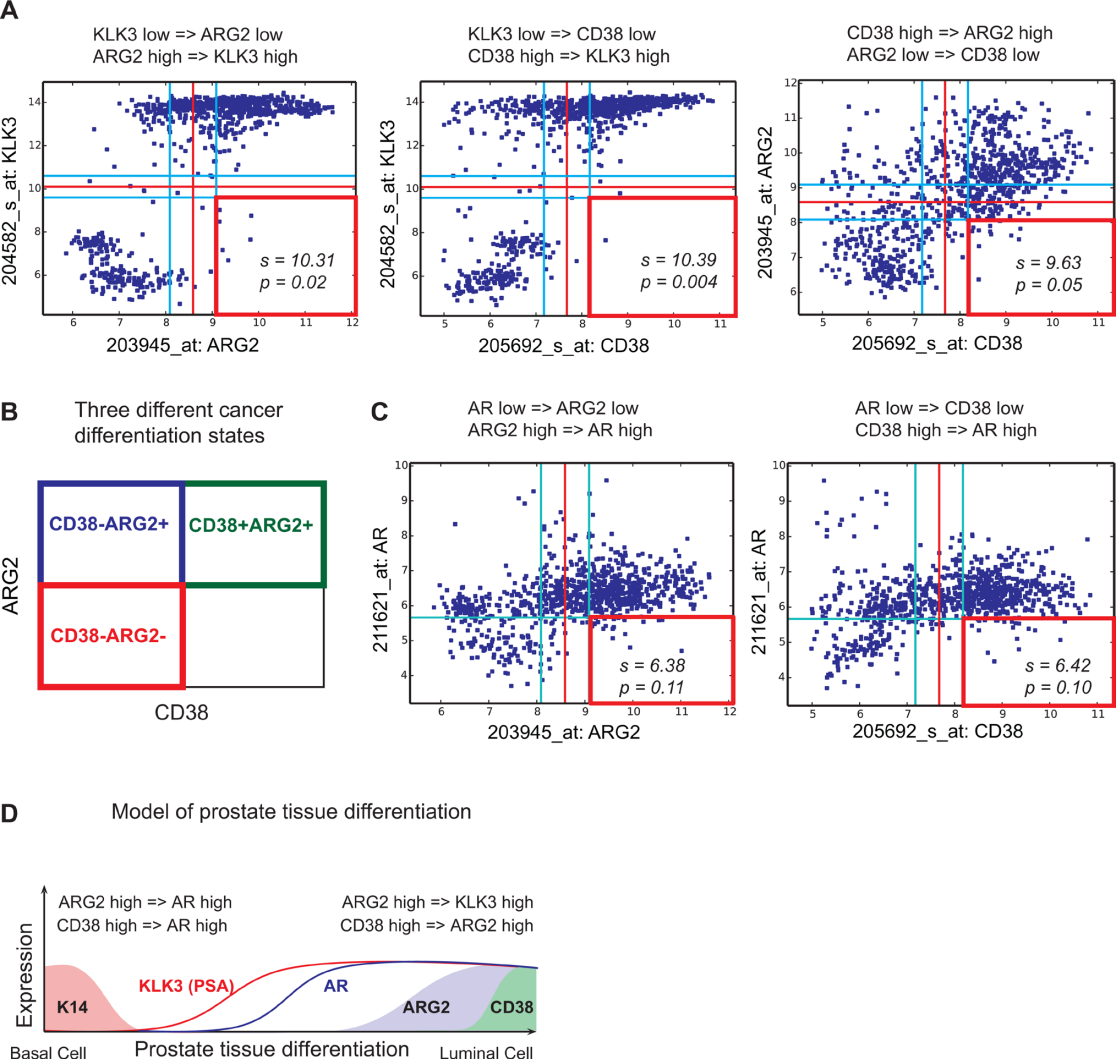
Relationship between KLK3, AR, ARG2 and CD38 Scatter plots showing relationships between KLK3 (PSA), ARG2 and CD38 in the Global-Prostate database containing 891 samples. Red lines indicate StepMiner threshold and light blue lines indicate noise margin around the threshold. Red square indicates a sparse quadrant with BooleanNet statistics s and p. We used a threshold of s > 3 and *p <* 0.1 for statistical significance. (**A**) KLK3 low => ARG2 low. KLK3 low => CD38 low. CD38 high => ARG2 high. (**B**) Three different prostate cancer differentiation states: CD38+ARG2+, CD38-ARG2+, and CD38-ARG2-. (**C**) ARG2 high => AR high. CD38 high => AR high. (**D**) A computational model of prostate tissue differentiation.

CD38 and ARG2 have a robust Boolean implication relationship with KLK3 and AR as shown in Figure 2. When ARG2 was compared with KLK3 in the Global-Prostate dataset, we observed that the ARG2 high and KLK3 low quadrant was significantly sparse (FDR < 1e-4), thereby satisfying criteria for a Boolean implication relationship. CD38 showed a similar Boolean relationship with KLK3 and with ARG2. A detailed investigation of the gene expression relationship between CD38 and ARG2 demonstrated Boolean relationship “CD38 high => ARG2 high” (Figure 2A) and three possible groupings of prostate cancer cases: CD38+ARG2+, CD38-ARG2+, and CD38-ARG2- (Figure 2B). We investigated the relationship between AR, CD38 and ARG2. Since the AR mRNA expression range was small, we relaxed the threshold (use t-0.5 as the threshold) and perform analysis without ignoring the noise zone as shown in Figure 2C. This analysis revealed a “CD38 high => AR high” and “ARG2 high => AR high” Boolean relationship. Both AR and PSA (KLK3) were expressed at low levels in basal cells and progressively expressed at high levels as cells are differentiated. Together with this differentiation related expression patterns of AR and KLK3 and the Boolean relationship between CD38, ARG2, AR and KLK3, the best possible scenario for ARG2 and CD38 expression pattern can be derived as shown in Figure 2D. ARG2 expression is hypothesized to turn on earlier compared to CD38 expression along the path of differentiation which is consistent with “CD38 high => ARG2 high”. From this analysis, we hypothesized that differentiation proceeds from CD38-ARG2- (least differentiated), to CD38-ARG2+ (moderately differentiated) and finally CD38+ARG2+ (most differentiated) (Figure 2D).

**Figure 3:**
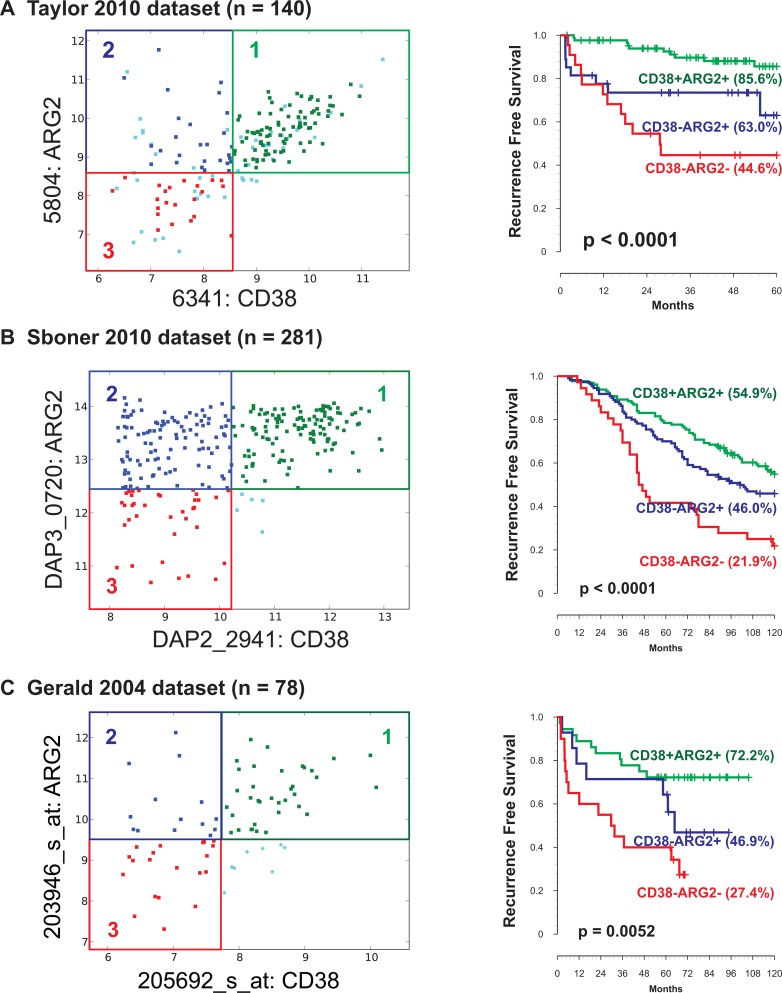
Association of ARG2 and CD38 transcript levels with patient outcome Three prostate cancer differentiation states ARG2+CD38+, ARG2+CD38-, and ARG2-CD38- were identified in three independent datasets. (**A**) Taylor-2010 dataset (*n =* 140). (**B**) Sboner-2010 dataset (*n =* 281). (**C**) Gerald-2004 dataset (*n =* 78). In all datasets (total *n =* 499), CD38-ARG2- groups were associated with lowest, CD38-ARG2+ groups were associated with moderate, and CD38+ARG2+ groups were associated with highest 10-year recurrence-free survival.

As expected, Boolean analysis on these three datasets confirmed the existence of three differentiation states: CD38+ARG2+, CD38-ARG2+, and CD38-ARG2-. Cases were grouped using these criteria, and 10-year recurrence-free survival was compared each of the 3 groups. In the Taylor *et al.* dataset, Kaplan–Meier analysis demonstrated that recurrence after surgery occurred in the expected order with CD38-ARG2- showing the highest risk of recurrence, CD38-ARG2+ intermediate risk and CD38+ARG2+ showing the lowest rates of recurrence (Figure 3A, *p <* 0.001). We observed similar results in Sboner-2010 dataset (Figure 3B, *p <* 0.001) and Gerald-2004 dataset (Figure 3C, *p <* 0.01).

### Association of CD38 and ARG2 protein expression with clinical and pathological features

**Figure 4:**
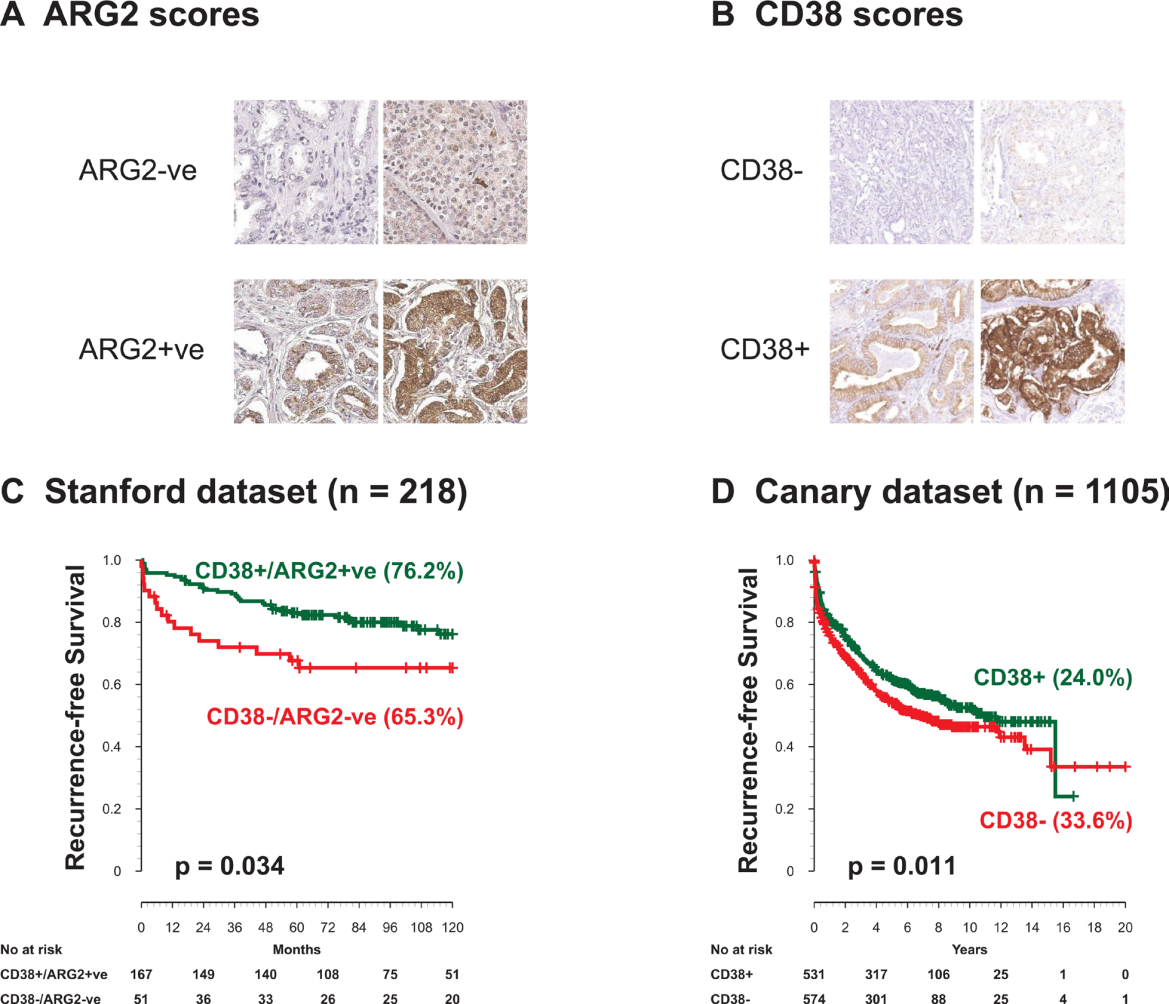
ARG2 and CD38 protein levels by IHC and outcome after surgery ARG2 and CD38 protein expression levels were evaluated in two independent cohorts. (**A**) representative staining of ARG2. (**B**) Representative staining of CD38. (**C**) In the Stanford TMA dataset, CD38-ARG2- cancer staining shows significantly lower 10-year recurrence-free survival compared to CD38+ARG2+ groups. (**D**) The Canary dataset containing 1105 patients, showed CD38 expressing tumors had significantly higher 10-year recurrence-free survival. ARG2 expression in the Canary dataset was not associated with recurrence-free survival.

Expression of CD38 and ARG2 proteins was tested by IHC and associated with patient outcome using 2 tissue microarray sets representing independent patient datasets. Scoring strategies for CD38 and ARG2 are summarized in Figure 4A, 4B, Supplementary Figures 2, 3, and 4. In the Stanford-TMA dataset, we segregated cases into two groups of patients: CD38-ARG2- and CD38+/ARG2+ as described in the method section. Neither CD38 nor ARG2 was significantly associated with outcome when analyzed individually (Supplementary Figure 5A and 5B). When the biomarkers were used to create a combined score, we observed that the rate of 10-year RFS for CD38+/ARG2+ group was significantly higher compared to CD38-ARG2- group (Figure 4C, Supplementary Figure 5C, *p <* 0.05). However, this association was not significantly associated with RFS after adjusting for other clinical factors in the model such as grade, age and stage.

Since the Stanford-TMA cohort included 234 patients, it was possible that this set was underpowered to test whether CD38 and ARG2 protein expression was independent of clinical and pathological variables. We therefore assessed expression of ARG2 and CD38 in the context of clinical and pathological features using the Canary-TMA which includes over 1300 cases from 7 institutions. In this cohort, decreased CD38 protein expression by IHC was associated with decreased recurrence free survival in Kaplan–Meier analysis (Figure 4D, Supplementary Figure 6A, *p <* 0.05, Log-rank test). In addition, negative/weak CD38 expression was significantly associated with adverse pathological features including seminal vesicle invasion (Table 1, SVI, *P =* 0.01, Fisher’s exact test) and extracapsular extension (Table 1, ECE, *P =* 0.02, Fisher’s exact test). CD38 expression did not correlate with age, pre-operative serum PSA levels, positive surgical margins (PSM) or Gleason score (GS), although there was a trend for lower expression associated with higher Gleason score (Supplementary Table 2). Univariable Cox proportional hazard model analysis showed that negative/weak CD38 staining by IHC was significantly associated with worse RFS (Table 2, *p* = 0.01), as were PSM, SVI, ECE, higher GS, and higher pre-operative PSA. However, CD38 IHC was not significantly associated with RFS, OS, or DSS after adjusting for other clinical factors in the multivariate analysis (Supplementary Table 3). In the Canary-TMA cohort, ARG2 protein level neither alone nor in combination with CD38 was associated with RFS, DSS, OS or any of the clinical and pathological variables on univariable and multivariable analysis (Supplementary Figure 6B).

**Table 1:** Summary of margin, SVI, ECE, and Gleason by CD38 IHC status

	CD38 IHC					All	
	Moderate/Strong		Negative/Weak		*P*-value		
	*N*	%	*N*	%		*N*	%
**Margin**							
**Missing**	77	50.99	74	49.01		151	13.67
**Positive**	159	46.9	180	53.1	0.75	339	30.68
**Negative**	295	47.97	320	52.03		615	55.66
**SVI**							
**Missing**	12	75	4	25		16	1.45
**No**	495	48.67	522	51.33	0.01	1017	92.04
**Yes**	24	33.33	48	66.67		72	6.52
**ECE**							
**Missing**	4	30.77	9	69.23		13	1.18
**No**	383	50.66	373	49.34	0.02	756	68.42
**Yes**	144	42.86	192	57.14		336	30.41
**Gleason**							
**Missing**	4	50	4	50		8	0.72
**<=6**	226	49.34	232	50.66	0.1	458	41.45
**3+4**	207	50.49	203	49.51		410	37.1
**4+3**	52	42.98	69	57.02		121	10.95
**10-Aug**	42	38.89	66	61.11		108	9.77
**All**	531	48.05	574	51.95		1105	100

**Table 2:** Univariable cox proportional hazard model of outcomes by clinical and pathological features and CD38 expression

Endpoint	Factor	Comparison	Hazard ratio	95% LCL	95% UCL	*P*-value	^#^Event	^#^Censored	Total ^#^patients
RFS	CD38 IHC	Moderate/Strong vs. Negative/Weak	0.796	0.668	0.949	0.01	507	598	1105
	Margin	Pos. vs. Neg.	2.135	1.767	2.581	<0.0001	431	523	954
	SVI	Yes vs. No	0.31	0.235	0.408	<0.0001	496	593	1089
	ECE	Yes vs. No	0.527	0.441	0.63	<0.0001	500	592	1092
	Gleason	3+4 vs. <=6	1.324	1.073	1.634	0.01	500	597	1097
		4+3 vs. <=6	2.265	1.733	2.961	<0.0001			
		8–10 vs. <=6	2.345	1.77	3.106	<0.0001			
	Age	1 year increase	1.004	0.991	1.016	0.56	488	515	1003
	Log (Pre-op PSA)	1 unit increase	1.91	1.651	2.209	<0.0001	461	525	986
OS	CD38 IHC	Moderate/Strong vs. Negative/Weak	0.75	0.434	1.297	0.3	53	1043	1096
	Margin	Pos. vs. Neg.	1.517	0.874	2.632	0.14	51	897	948
	SVI	Yes vs. No	0.456	0.205	1.011	0.053	52	1029	1081
	ECE	Yes vs. No	0.723	0.413	1.266	0.26	51	1032	1083
	Gleason	3+4 vs. <=6	0.816	0.398	1.673	0.58	53	1035	1088
		4+3 vs. <=6	1.517	0.606	3.798	0.37			
		8–10 vs. <=6	3.969	2.024	7.785	0.0001			
	age	1 year increase	1.07	1.026	1.115	0.0017	53	941	994
	Log (Pre-op PSA)	1 unit increase	1.65	1.087	2.505	0.02	35	942	977
DSS	CD38 IHC	Moderate/Strong vs. Negative/Weak	0.848	0.49	1.466	0.55	52	1048	1100
	Margin	Pos. vs. Neg.	2.796	1.429	5.47	0.0027	36	915	951
	SVI	Yes vs. No	0.293	0.147	0.584	0.0005	52	1033	1085
	ECE	Yes vs. No	0.513	0.294	0.896	0.02	50	1037	1087
	Gleason	3+4 vs. <=6	2.163	1.027	4.552	0.04	51	1041	1092
		4+3 vs. <=6	2.87	1.108	7.434	0.03			
		8-10 vs. <=6	6.513	2.945	14.403	<0.0001			
	age	1 year increase	1.028	0.988	1.07	0.18	51	947	998
	Log (Pre-op PSA)	1 unit increase	2.325	1.628	3.321	<0.0001	46	935	981

Hazard ratio higher than 1 means worse prognosis. LCL = lower confidence limit, UCL = upper confidence limit.

RFS: Recurrence Free Survival; OS: Overall Survival; DSS: Disease Specific Survival

## DISCUSSION

We used Boolean logic to identify genes whose expression correlates with the androgen signaling axis, a pathway activated in terminally differentiated prostate luminal cells. We identified loss of expression of CD38 protein as a prognostic biomarker that correlates with several features of aggressive prostate cancer including advanced stage (T3 including both SVI and ECE) and RFS. Our data clearly demonstrate that CD38 and ARG2 identify three different differentiation states in prostate cancer. There is a robust Boolean pattern that relates the expression of CD38 and ARG2; namely, when CD38 expression levels are high, ARG2 expression levels are also high. The findings in the current study, coupled with our work in bladder and colon cancer and myeloid cell development [10–12], strongly suggest that application of Boolean analysis to large gene expression datasets can provide biological insights and define new clinically relevant prognostic biomarkers.

There have been a few relatively small studies of CD38 in the normal and malignant prostate. A graded decrease in CD38 protein expression has been observed in 23 prostate samples comparing normal prostate glands distant from cancer with normal glands adjacent to cancer, and with prostate cancer glands [27]. Heterogeneous loss of CD38 in prostate cancer samples compared to normal prostate tissues has been observed in a broad survey of cell surface (CD) marker expression [28]. The functional consequences of low CD38 expression have been explored only recently. Low CD38 transcript levels are part of a set of 91 transcripts that define a basal/stem cell signature in prostate epithelial cells and this signature is enriched in aggressive and neuroendocrine-type castrate resistant prostate cancers [29]. In a follow-up study, Liu *et al.* demonstrated that luminal cells with low CD38 expression are enriched in normal prostate acini adjacent to inflammation and these cells have progenitor-like features. Isolated cells expressing low levels of CD38 display increased expression of inflammatory genes, generate significantly more organoids than high CD38 expressing cells, and can generate normal prostate glands and carcinomas in an *in vivo* reconstitution mouse model [30]. Chronic inflammation in the prostate has been associated prostate carcinogenesis and with decreased AR expression in luminal cells, potentially linking inflammation to the Boolean relationship we have observed between CD38 and AR [31]. In addition, the finding that low CD38 protein expressing cells have a progenitor-like phenotype confirms the ability of Boolean analysis to identify genes expressed in differentiation pathways in the prostate as we have observed in other tissue types [10–12].

Less is known about the role of ARG2 in prostate cancer. ARG2 expression has been reported to be relatively higher in normal and non-malignant prostatic tissues compared to prostate cancer tissues [32]. ARG2 expression is also androgen regulated and has been linked to immunosuppressive pathways in human prostate cancer [32]. In agreement with this observation, deletion of the ARG2 leads to increased tumor size in the TRAMP mouse model of prostate cancer [33]. These findings confirm the Boolean relationship of ARG2 with AR signaling. The finding that the percentage of ARG2 staining was prognostic in the Stanford-TMA cohort suggests that ARG2 expression could be a marker of differentiation and that its loss correlates with more aggressive prostate cancer. The lack of validation in the Canary-TMA cohort could be due to differences in the scoring procedures used that confounded the association of ARG2 with clinical behavior. It is possible that re-evaluation and optimization of ARG2 scoring, possibly using quantitative imaging analysis approaches, will improve performance of this biomarker.

Our findings confirm that Boolean analysis approaches can be used to identify markers of differentiation that have biological and clinical relevance. Specifically, we have identified CD38 as a marker of differentiation in prostate cancer and confirmed that decreased of expression of CD38 transcripts and protein by IHC is associated with aggressive prostate cancer. These findings agree with recent observations demonstrating that CD38 loss correlates with a basal/progenitor class of luminal cells. As more markers of progenitor and stem cells are identifies in the prostate, Boolean approaches could yield additional genes relevant to prostate differentiation and as clinical biomarkers of prognosis.

## METHODS

### Gene expression datasets

Publicly available prostate cancer gene expression datasets with associated clinical information were downloaded from National Center for Biotechnology Information (NCBI) Gene Expression Omnibus website (GEO) and European Bioinformatics Institute ArrayExpress [34–36] as described in Supplementary Table 1A. A large global prostate cancer microarray database (Global-Prostate, *n =* 891) was created from Human U133A (GPL96), Human U133 Plus 2.0 (GPL570), and Human U133A 2.0 (GPL571) Affymetrix platforms as described in Supplementary Table 1B. Gene expression values for each Affymetrix platform were normalized by robust multichip average (RMA) algorithm [37]. Three independent publicly available prostate cancer datasets were annotated with recurrence-free survival: Gerald-2004 (*n =* 78, Memorial Sloan-Kettering Cancer Center) [23], Taylor-2010 (*n =* 367, Memorial Sloan-Kettering Cancer Center) [25], and Sboner-2010 (*n =* 281, Swedish Watchful Waiting cohort) [24].

### Boolean analysis of datasets

The expression values of each gene were ordered from low to high and a rising step function was computed to define a threshold t by the StepMiner algorithm in each individual dataset [38]. If the assigned threshold for a gene was t, then expression levels above t+0.5 were classified as “high”, and the expression levels below t-0.5 were classified as “low”. Expression values between t -0.5 and t +0.5 were classified as “intermediate” (Supplementary Figure 1A). The previously published BooleanNet algorithm was used to determine Boolean Implication relationships between genes (Supplementary Figure 1B) [39]. Briefly, BooleanNet algorithm searches for at least one sparsely populated quadrant in a scatterplot between two genes. The “intermediate” expression values are ignored by the BooleanNet algorithm. There are six possible scenarios: one of the four quadrants is sparse (four independent asymmetric Boolean implications) or two diagonally opposite quadrants are sparse (Equivalent and Opposite Boolean implications).

### Stanford tissue microarray (TMA) resource

All samples used in the construction of the TMA were used only for men who signed an IRB-approved Informed Consent for use of their tissues samples. A tissue arrayer (Beecher Instruments, Sun Prairie, WI) was used to construct a prostate cancer tissue microarray (Stanford-TMA) comprising an independent set of 234 formalin-fixed, paraffin-embedded primary prostate tumor cases selected from radical prostatectomy specimens collected at Stanford University, with institutional review board approval. Duplicate 0.6 mm tumor cores represented each case, and the series was associated with a minimum clinical follow-up of 5 years and a median follow-up of 8 years.

### The Canary prostate cancer TMA resource

Tissue blocks and accompanying clinical data were collected at each of the participating sites (Stanford University, University of California San Francisco, University of Washington, University of British Columbia, University of Texas Health San Antonio, Eastern Virginia Medical School) under a research protocol developed by the investigators with IRB approval at each institution. The approved protocols included sharing of de-identified data and samples and correlation of clinical data with biomarker data acquired from the TMAs. A materials transfer agreement was developed jointly and approved at each site for sharing of clinical data and tissue samples.

Testing of CD38 and ARG2 as clinical biomarkers of prognosis was carried out using tissue microarrays (TMA) comprised of 4 core samples from over 1300 randomly selected participants treated for PC with RP at six institutions between 1995 and 2004 [8]. The cohort includes approximately equal numbers of samples from men with biochemically recurrent and non-recurrent PC with 5 or more years of follow-up. The TMA (Canary-TMA) was constructed to assess biomarkers that provide prognostic information independent of clinical and pathological information. Patient characteristics were collected in the clinical data set and included pre-operative serum PSA level, pathology stage, Gleason score (GS), seminal vesicle invasion (SVI), extracapsular extension (ECE), and surgical margin status (positive or negative). The primary endpoint was post-surgery recurrence-free survival (RFS) from the date of surgery, where the survival event was defined as any prostate cancer recurrence (biological, clinical/radiological, or use of salvage therapy), metastasis, or prostate cancer death. Overall survival (OS) and Disease Specific Survival (DSS) were secondary endpoints.

### Immunohistochemistry (IHC)

Freshly cut 5 micron sections were obtained and immunohistochemistry was performed using a commercial antibody to CD38 (CD38-290-L-CE, 1:25, Leica (Novocastra)) by the Department of Pathology Immunodiagnostic Laboratory using standard optimized protocols. ARG2 expression was assessed by IHC using a commercial antibody (SC20151, 1:50, Santa Cruz Biotechnology Inc., Santa Cruz, CA). Cancer cores were scored on a 0–3 scale based on staining intensity where negative was 0, weak 1, moderate 2 and strong 3 (described in Supplementary Figure 2 and 3). In the Stanford cohort, ARG2 was scored based on the percentage of positively stained tumor cells as described in Supplementary Figure 4: 0 – 1% positive (score 0), 1 – 33% positive (score 1), 33 – 66% positive (score 2), 66 – 95% positive (score 3), and greater than 95% positive (score 4). Because of the large number of cases and cores, ARG2 percentage scoring was not performed in the Canary TMA cohort. The CD38 IHC score used in the analysis was the maximum score of all the cores from that patient and the ARG2 IHC was the minimum score of all the cores from that patient. Strong/moderate scores (score 3 and 2) were considered positive, and weak/negative scores (score 1 and 0) were considered negative. For the Stanford cohort, ARG2 percent was used. ARG2 negative (ARG2 –ve) score was computed by combining raw ARG2 percentage score 0, 1, and 2. ARG2 positive (ARG2 +ve) score is computed by combining raw ARG2 percentage score 3 and 4. In the Stanford cohort, 216 patients had high-quality CD38 staining and 219 patients had high-quality ARG2 staining available for analysis. A combined CD38/ARG2 score was generated such that patients were scored “CD38-/ARG2-ve” if the ARG2 score was 0 or 1 or ARG2 score was 2 and CD38 score was 0 or 1. Patients were scored “CD38+/ARG2+ve” if ARG2 score was 3 or 4 or ARG2 score was 2 and CD38 score was 2 or 3.

### Statistical analysis

Of 1326 patients with clinical data in the Canary cohort, 1105 patients had complete high-quality CD38 staining and 1122 patients had complete high-quality ARG2 staining available for analysis with acceptable strong uniform TMA staining of the positive controls. In this cohort, the scores were analyzed separately as prognostic variables since we did not have percentage ARG2 staining. Summary statistics of patients’ CD38 IHC score and other clinical factors (ECE, SVI, margin, Gleason score) were provided in frequencies and percentages (Table 1). Patient age and pre-op PSA were summarized using mean, SD, and range. Fisher’s exact test was used to assess correlations between CD38 IHC with other clinical factors. Wilcoxon rank sum test was used to compare age and pre-operative PSA between CD38 IHC groups. Cox proportional hazard models were used to assess effect of each factor and multiple factors on RFS, OS, and DSS. All tests were two-sided and p-values of 0.05 or less were considered statistically significant. Statistical analysis was carried out using SAS version 9 (SAS Institute, Cary, NC) and R version 3.2.3 (2015–12-10) — “Wooden Christmas-Tree”.

## SUPPLEMENTARY MATERIALS FIGURES AND TABLES


