# Supplementary material for: Boolean analysis identifies CD38 as a biomarker of aggressive localized prostate cancer
Source: Oncotarget. 2018 Jan 5;9(5):6550–61. doi: 10.18632/oncotarget.23973 (PMC5814231; doi:10.18632/oncotarget.23973)
Supplement: Supplementary file 1 [file oncotarget-09-6550-s001.pdf]

Boolean analysis identifies CD38 as a biomarker of aggressive localized prostate cancer

SUPPLEMENTARY MATERIALS

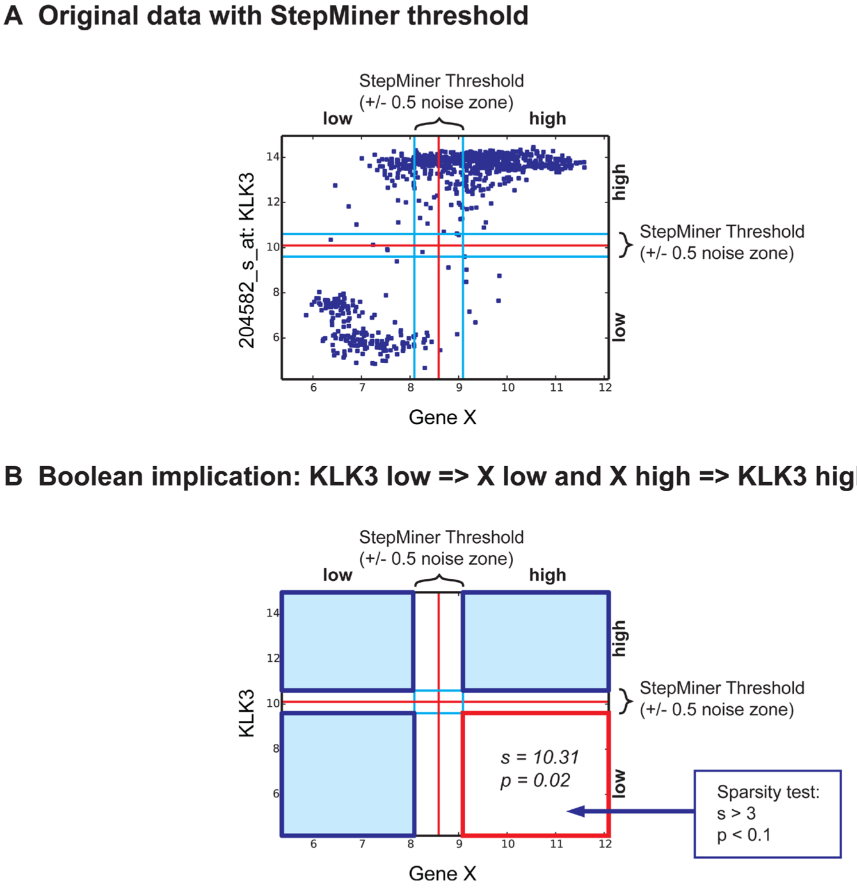

Supplementary Figure 1: Search for Boolean implication patterns: The method of Boolean analysis used on the global prostate cancer gene expression database containing 891 microarrays from diverse prostate tissues including prostate cancer, BPH, stroma, PIN, cell lines and normal prostate samples. Gene-expression levels were assigned for each gene in each array, using the log2 of the normalized expression values by RMA algorithm. (**A**) Scatter plot of gene expression values (log2 normalized) between KLK3 and a candidate gene X. The thresholds for definition of high and low values were calculated using the StepMiner algorithm and an intermediate region was defined around each threshold with a width of 1 (i.e. threshold +/– 0.5), corresponding to a 2-fold change in gene expression, which is the minimum noise level in these datasets (Sahoo *et al*., Genome Biology, 9:R157, 2008). All the data below the intermediate region (< StepMiner threshold - 0.5) were considered low, and all above the intermediate region (> StepMiner threshold + 0.5) were considered high. (**B**) Boolean implication relationship was identified between pairs of transcripts using the "Boolean-Net" software algorithm (Sahoo *et al*., Genome Biology, 9:R157, 2008). The algorithm searches for a sparse quadrant using a sparsity test (statistic: s > 3 and error probability: $p < 0.1$; for more aggressive thresholding $s > 10$, $p < 0.01$ was used) ignoring the intermediate values. In panel B the bottom right quadrant is sparse, thereby identifying a Boolean implication relationship between KLK3 and X: X high => KLK3 high. This is equivalent to KLK3 low => X low. The algorithm uses all possible candidate transcripts X to identify a list of genes that satisfy the Boolean implication.

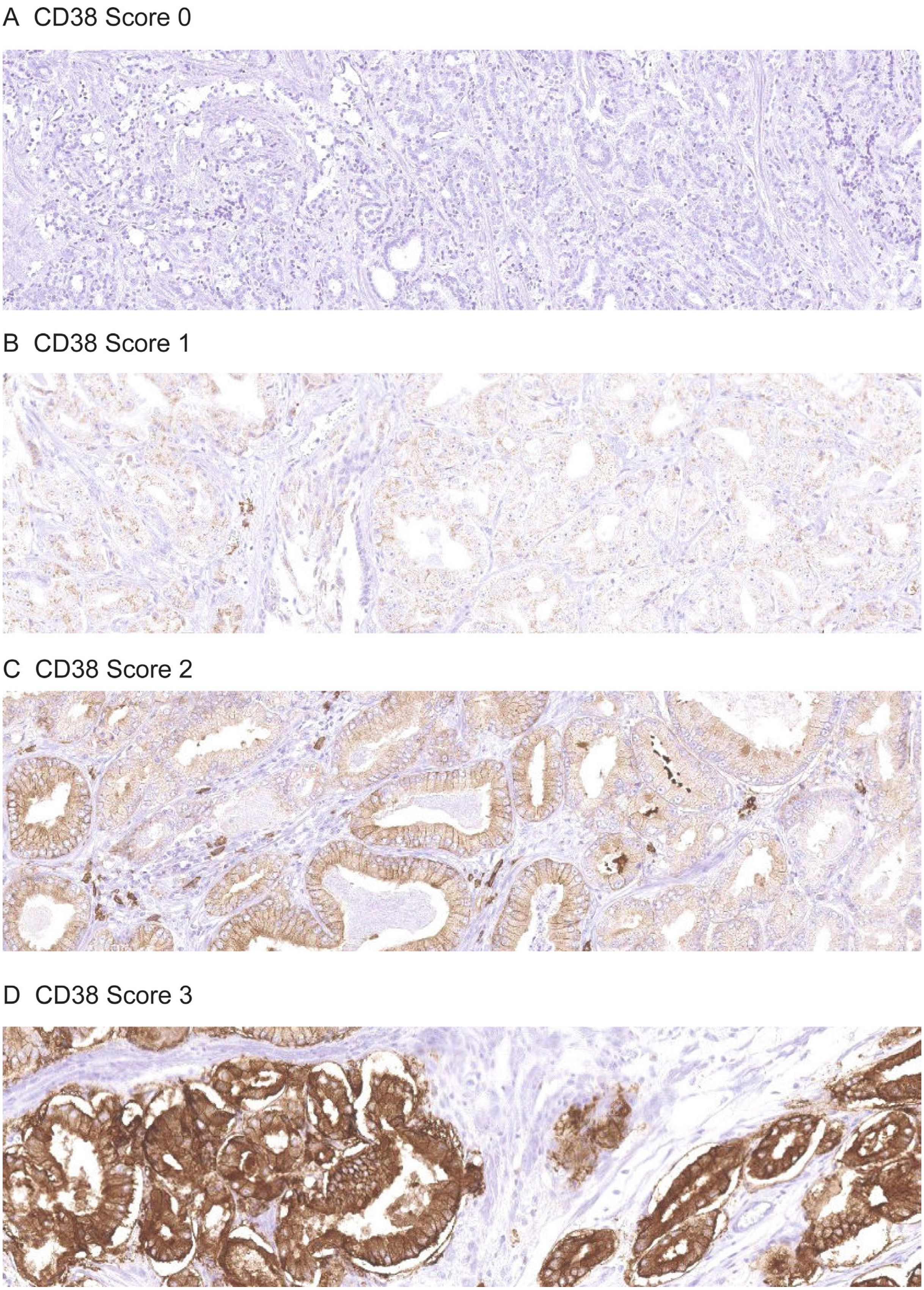

Supplementary Figure 2: Scoring CD38 based on Intensity. (**A**) CD38 negative cores show absent staining (Score = 0). (**B**) CD38 weak cores display weak uniform staining (Score = 1). (**C**) CD38 moderate cores with patches of strong and weak staining (Score = 2). (**D**) CD38 strong cores show uniform strong staining in all tumor cells (Score = 3).

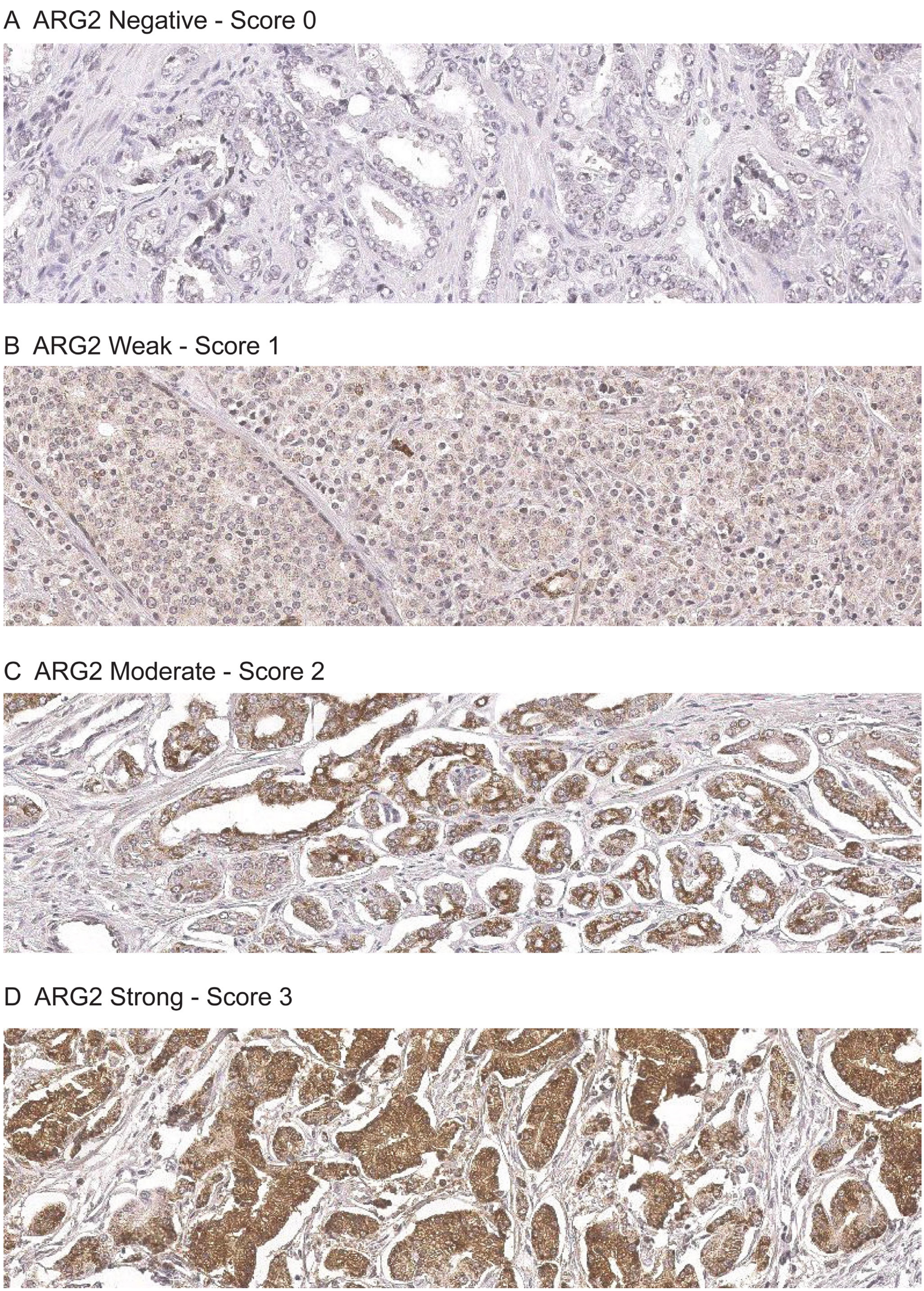

Supplementary Figure 3: ARG2 staining intensity scoring used on the Canary TMA. (**A**) ARG2 negative cores lack staining (Score = 0). (**B**) ARG2 weak cores show weak uniform staining (Score = 1). (**C**) ARG2 moderate cores display patchy strong and weak staining (Score = 2). (**D**) ARG2 strong cores show strong staining in all tumor cells (Score = 3).

A ARG2 Score 0 - all tumor cells negative

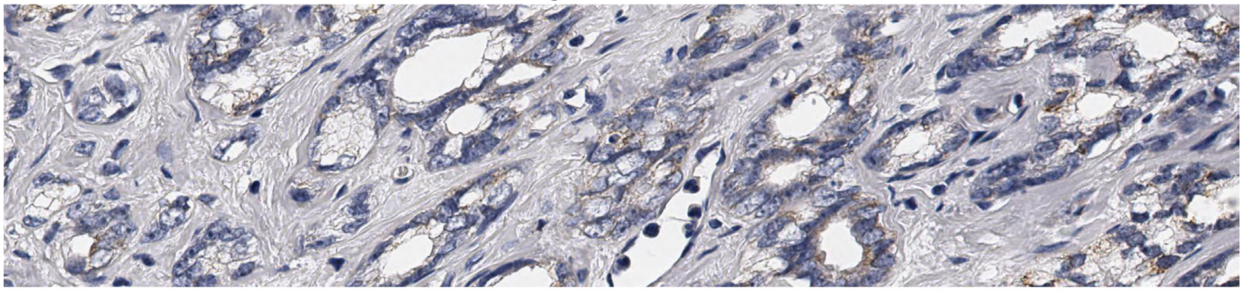

B ARG2 Score 1 - 1 to 33% tumor cells positive

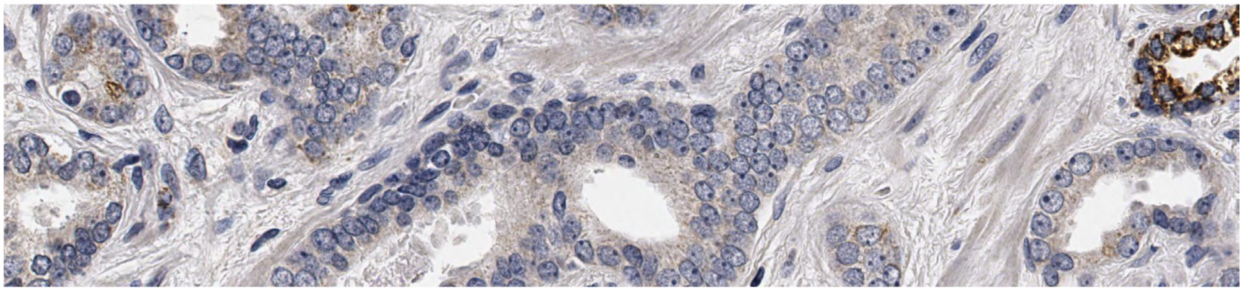

C ARG2 Score 2 - 33% to 66% tumor cells positive

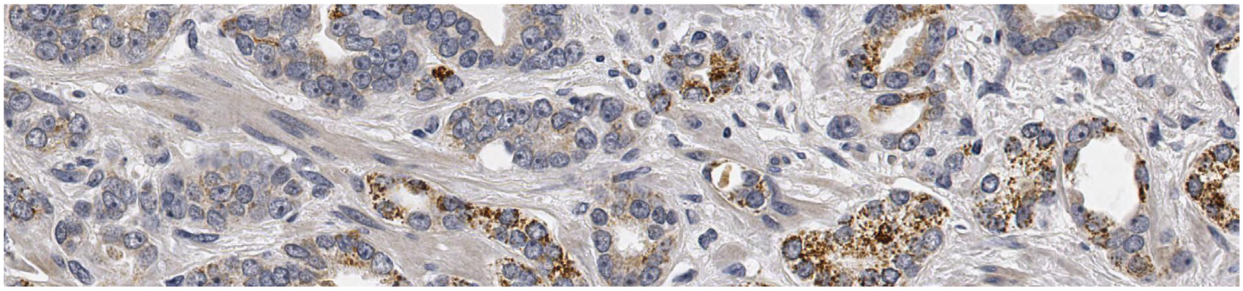

D ARG2 Score 3 - 66% to 95% tumor cells positive

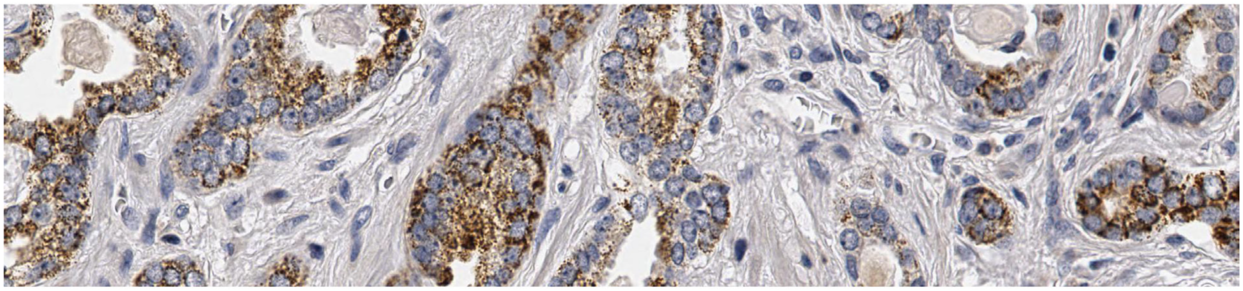

E ARG2 Score 4 - greater than 95% tumor cells positive

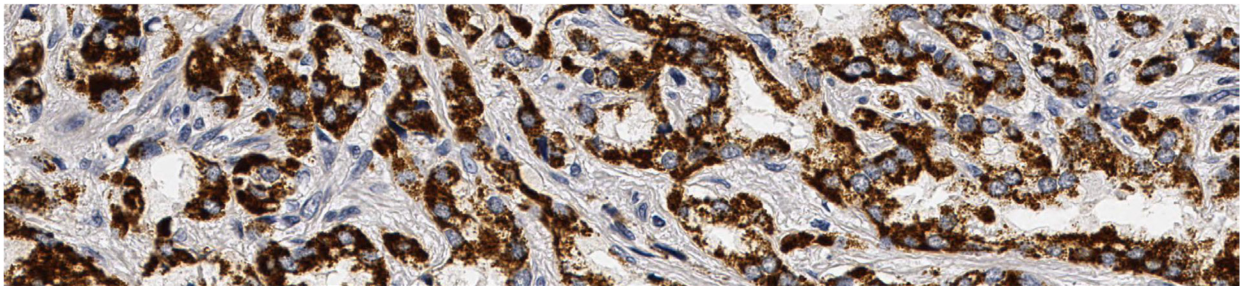

Supplementary Figure 4: Scoring based on percentage of ARG2 positive cells. ARG2 staining on the Stanford TMA was evaluated based on the percentage of ARG2 positive tumor cells. (**A**) Score was assigned 0 if all the tumor cells are negative. (**B**) Score was assigned 1 if there was 1 to 33% tumor cells positive. (**C**) Score was assigned 2 if there was 33% to 66% tumor cells positive. (**D**) Score was assigned 3 if there was 66% to 95% tumor cells positive. (**E**) Score was assigned 4 if more than 95% of tumor cells were positive.

A Kaplan-Meier analysis - CD38

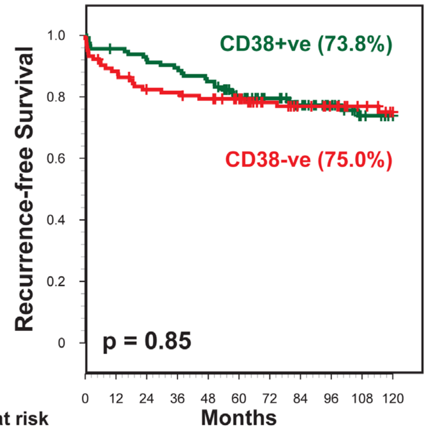

No at risk						
CD38+ve	114	104	96	74	51	34
CD38-ve	103	82	78	61	50	38

B Kaplan-Meier analysis - ARG2

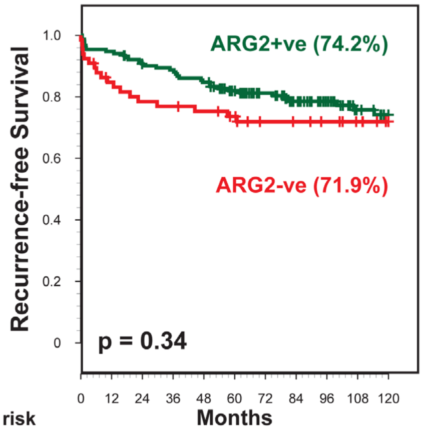

No at risk						
ARG2+ve	152	135	126	95	65	43
ARG2-ve	66	50	47	39	35	28

C Prognostic power of CD38 and ARG2 combined

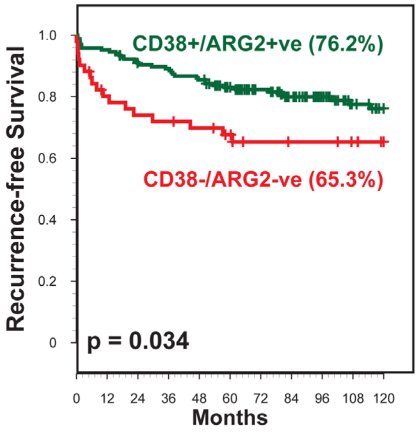

No at risk						
CD38+/ARG2+ve	167	149	140	108	75	51
CD38-/ARG2-ve	51	36	33	26	25	20

D Multivariate analysis of the combined CD38 and ARG2 groups

	HR	95% CI	p	c	HR	95% CI	p	c
groups	1.86	1.04 - 3.32	0.037	*	1.26	0.69 - 2.30	0.45	
grade	4.9	2.83 - 8.47	1.30E-08	***	3.34	1.88 - 5.92	3.70E-05	***
age	0.99	0.95 - 1.03	0.46		0.97	0.93 - 1.01	0.12	
stage	6.27	3.87 - 10.15	8.20E-14	***	5.53	3.28 - 9.31	1.40E-10	***

Supplementary Figure 5: Stanford TMA cohort analysis. (**A**) Kaplan-Meier analysis of CD38 scores shows that CD38 scores were not significantly associated with 10 year recurrence-free survival (RFS). (**B**) Kaplan-Meier analysis showing that ARG2 scores are not significantly associated with RFS. (**C**) Combined CD38 and ARG2 scores correlate with RFS. (**D**) Multivariable analysis of CD38/ARG2 staining and RFS.

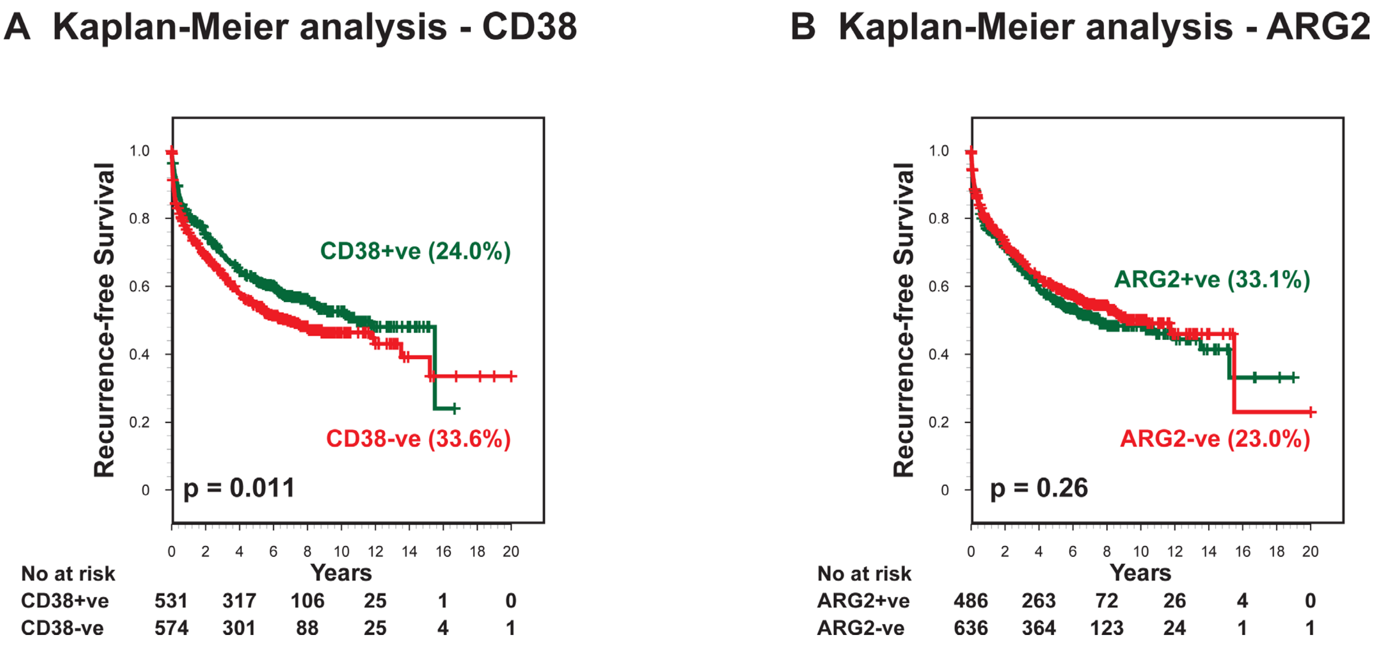

Supplementary Figure 6: Canary cohort analysis. (**A**) Kaplan–Meier analysis of CD38 scores in the Canary tissue microarray shows that CD38 scores are significantly associated with 10 year recurrence-free survival ($p = 0.011$). (**B**) Kaplan–Meier analysis of ARG2 scores in the Canary tissue microarray shows that ARG2 scores are not significantly associated with 10 year recurrence-free survival ($p = 0.26$).

Supplementary Table 1: List of prostate cancer datasets

A. Prostate cancer datasets

Name	Journal	Year	Pubmed	RAW	GEO/AE	Platform	Survival	#patients
Singh D	Cancer Cell	2002	12086878	yes	NA	U95Av2	no	102
Glinsky GV	J Clin Invest.	2004	15067324	yes	NA	U133A2	yes	78
Lapointe J	PNAS	2004	14711987	yes	GSE3933	cDNA	yes	112
Gulzar	Oncogene	2010	22349817	yes	NA	HEEBO	yes	131
Chandran UR	BMC Cancer	2007	17430594		GSE6919	HG_ U95Av2	no	171
Pressinotti NC	Mol Cancer	2010	20035634		GSE15484	GPL3050	no	65
Sboner A	BMC Med Genomics	2010	20233430	yes	GSE16560	GPL5474	yes	281
Wang Y	Cancer Res	2009	20663908	yes	GSE17951	U133Plus2	no	154
Taylor BS	Cancer Cell	2010	20579941	yes	GSE21034	HuEx-1_0-st	yes	367
Setlur	J Natl Cancer Inst	2008	18505969	yes	GSE8402	GPL5474	no	472

B. Global prostate cancer database

Name	Journal	Year	Pubmed	RAW	GEO/AE	Platform	Survival	#patients
Bakshi S	Environ Health Perspect	2008	18560533	yes	GSE9951	GPL570		19
Berry PA	Prostate	2011	21432868	yes	E-MTAB-402	GPL570		14
Best CJ	Clin Cancer Res	2005	16203770	yes	GSE2443	GPL96		20
Birnie R	Genome Biol.	2008	18492237	yes	E-MEXP-993	GPL570		36
Chambers KF	J Biomed Sci	2011	21696611	yes	E-MEXP-2034	GPL570		40
Guyon I		2011		yes	E-TABM-456	GPL96		85
Liu P	Cancer Res	2006	16618720	yes	E-TABM-26	GPL96		57
Sun Y	Prostate	2009	19343730	yes	GSE25136	GPL96		79
Traka M	PLoS One.	2008	18596959	yes	E-MEXP-1243	GPL570		81
Tsavachidou D	J Natl Cancer Inst.	2009	19244175	yes	E-MEXP-1327	GPL96		85
Varambally S	Cancer Cell	2005	16286247	yes	GSE3325	GPL570		19
Wallace TA	Cancer Res	2008	18245496	yes	GSE6956	GPL571		72
Wang Y	Cancer Res	2010	20663908	yes	GSE8218	GPL96		130
Wang Y	Cancer Res	2010	20663908	yes	GSE17951	GPL570		154

Panel A shows a list of publicly available prostate cancer datasets with clinical information (Only five dataset with survival outcome). Panel B shows a list of prostate cancer datasets on Affymetrix U133A (GPL96), U133A 2.0 (GPL571) or U133 Plus 2.0 (GPL570) microarray platforms that are normalized together to build a large global prostate cancer database. The lists include the first author, journal where it was first published, year in which it was published, the PubMed id, GEO/ArrayExpress id, microarray platforms, survival annotation, and number of patients.

Supplementary Table 2: Summary of age and pre-op PSA by CD38 IHC status

1. No significant difference between CD38 IHC groups with respect to age or pre-op PSA								
		N	Mean	SD	Min	Median	Max	*P*-value
	**CD38 IHC**							
**age**	**Moderate/Strong**	489	61.37	6.84	42	62	78	0.72
	**Negative/Weak**	514	61.51	7.18	35	62	77	
	**All**	1003	61.44	7.02	35	62	78	
	**CD38 IHC**							
**Pre-op PSA**	**Moderate/Strong**	474	8.13	6.37	0.1	6.5	72.7	0.69
	**Negative/Weak**	512	8.99	9.91	0.84	6.4	124	
	**All**	986	8.57	8.4	0.1	6.41	124	

P-values by Wilcoxon rank sum test.

Supplementary Table 3: Multivariate cox proportional hazard model of outcomes

Endpoint	Factor	Comparison	Hazard Ratio	95% LCL	95% UCL	*P*-value
RFS	CD38 IHC	Moderate/Strong vs. Weak/Negative	0.827	0.671	1.018	0.07
	Margin	Pos. vs. Neg.	1.685	1.353	2.099	<.0001
	SVI	Yes vs. No	2.256	1.623	3.138	<.0001
	ECE	Yes vs. No	1.287	1.028	1.612	0.03
	Gleason	3+4 vs. 6	1.207	0.941	1.549	0.14
		4+3 vs. 6	1.919	1.407	2.617	<.0001
		8–10 vs. 6	1.522	1.094	2.118	0.01
	Log(pre-op PSA)	1 unit increase	1.437	1.219	1.695	<.0001
OS	CD38 IHC	Moderate/Strong vs. Weak/Negative	0.787	0.454	1.366	0.39
	Gleason	3+4 vs. 6	0.78	0.38	1.602	0.5
		4+3 vs. 6	1.362	0.542	3.421	0.51
		8–10 vs. 6	3.105	1.566	6.155	0.001
	Age	1 year increase	1.059	1.015	1.104	0.01
DSS	CD38 IHC	Moderate/Strong vs. Weak/Negative	0.941	0.516	1.715	0.84
	Gleason	3+4 vs. 6	2.296	0.99	5.323	0.053
		4+3 vs. 6	2.865	1.021	8.035	0.046
		8–10 vs. 6	5.476	2.175	13.788	0.000
	Log(pre-op PSA)	1 unit increase	1.985	1.351	2.917	0.001

Hazard ratio higher than 1 means worse prognosis. LCL = lower confidence limit, UCL = upper confidence limit.
RFS: Recurrence Free Survival; OS: Overall Survival; DSS: Disease Specific Survival.
